# Antidepressant-like effects of trophic factor receptor signaling

**DOI:** 10.3389/fnmol.2022.958797

**Published:** 2022-08-23

**Authors:** Monica Sathyanesan, Samuel S. Newton

**Affiliations:** ^1^Division of Basic Biomedical Sciences, Sanford School of Medicine, University of South Dakota, Vermillion, SD, United States; ^2^Sioux Falls VA Healthcare System, Sioux Falls, SD, United States

**Keywords:** neurotrophic factor, trophic factor, molecular structure, antidepressant, receptor signaling

## Abstract

A significant body of research has demonstrated that antidepressants regulate neurotrophic factors and that neurotrophins themselves are capable of independently producing antidepressant-like effects. While brain derived neurotrophic factor (BDNF) remains the best studied molecule in this context, there are several structurally diverse trophic factors that have shown comparable behavioral effects, including basic fibroblast growth factor (FGF-2), insulin-like growth factor 1 (IGF-1) and vascular endothelial growth factor (VEGF). In this review we discuss the structural and biochemical signaling aspects of these neurotrophic factors with antidepressant activity. We also include a discussion on a cytokine molecule erythropoietin (EPO), widely known and prescribed as a hormone to treat anemia but has recently been shown to function as a neurotrophic factor in the central nervous system (CNS).

## Introduction

Neurotrophic factors are a family of nervous system proteins that play essential roles in neuronal development, survival, plasticity and function. The neurotrophic factor hypothesis of depression and antidepressant action continues to be highly influential in the field of biological psychiatry, providing a firm molecular neurobiology framework for the behavioral actions of antidepressants. Over the years since its inception, supportive evidence has steadily accrued. Neurotrophic factors have been implicated in the molecular and behavioral actions of multiple classes of antidepressants, including selective serotonin reuptake inhibitors (Nibuya et al., 1996; Bath et al., 2012), electroconvulsive seizure (ECS; Nibuya et al., 1995; Newton et al., 2003), exercise (Hunsberger et al., 2007) and ketamine (Autry et al., 2011). Although the focus has been mainly on BDNF, there are several other neurotrophic factors that have shown antidepressant-like effects in preclinical studies. The substantial diversity in the structure of these molecules, receptors that they bind and intracellular signaling cascades that are activated, makes it worthwhile to examine the different trophic factors that have been demonstrated to produce behavioral effects. From a cellular neurobiology standpoint, few molecules can rival the potency of neurotrophic factors.

Despite a robust body of available evidence indicating that trophic molecules are regulated by antidepressants and are central to antidepressant activity and independently produce antidepressant effects, the findings have not led to the successful development of drugs with a primary neurotrophic mechanism of action. In this review, we start with an examination of BDNF and then discuss other trophic factors that have shown comparable antidepressant effects. Our discussion is limited to the trophic factors with well characterized receptors and signaling mechanisms. Behavioral studies that we discuss are primarily those that report antidepressant-like effects of independent trophic factors in rodent studies.

## Review

### Role of brain derived neurotrophic factor in antidepressant activity

**Figure 1:**
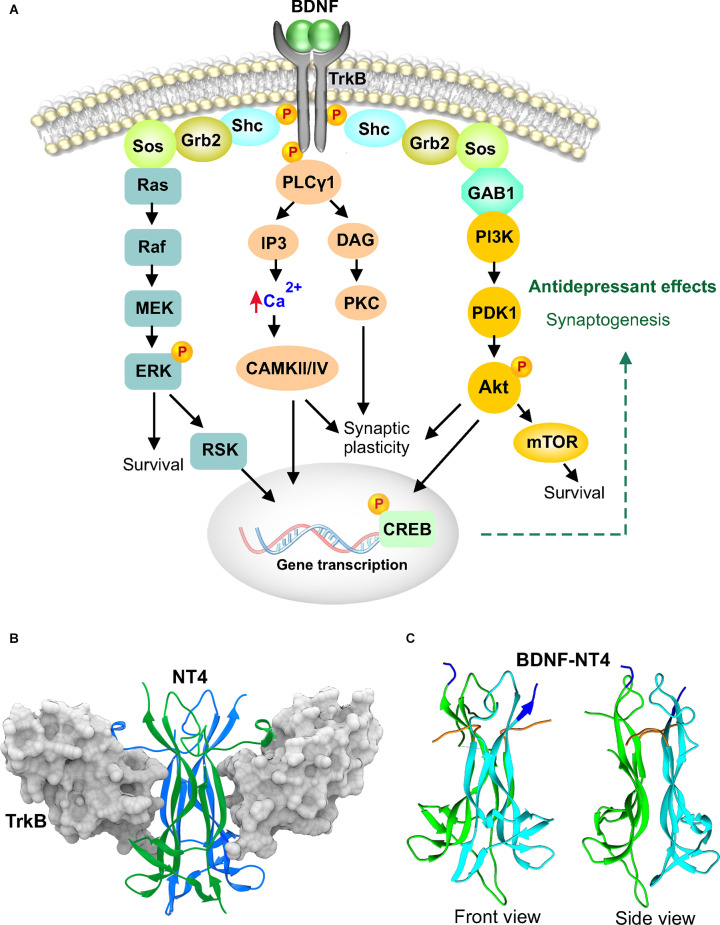
Brain derived neurotrophic factor (BDNF) signaling and structure. **(A)** BDNF binds as a dimer to the tropomyosin-related kinase B (TrkB) extracellular domain causing receptor dimerization, phosphorylation and activation. TrkB recruits and phosphorylates phospholipase C (PLCγ1) and Src homologous and collagen-like (Shc) adaptor molecule. Shc associates with growth factor receptor bound 2 (Grb2) and son of sevenless (SOS). This can lead to activation of the Ras-Erk pathway or association with Grb2-associated binder 1 (GAB1) to activate the PI3K-Akt-mTOR pathway. Activated TrkB can also recruit phospholipase C (PLCγ1) which hydrolyzes phospholipids to yield diacylglycerol (DAG) and inositol 1,4, 5-trisphosphate (IP3). DAG then activates protein kinase C (PKC) while IP3 mobilizes calcium. The released calcium activates Ca^2+^/calmodulin-dependent kinase (CAMKII, CAMKIV) which in turn activates the transcription factor, c-AMP response element binding protein (CREB) by phosphorylation. **(B)** Crystal structure of NT4 dimer bound to the extracellular TrkB domain 5, PDB 1HCF. The two NT4 molecules are shown as green and blue colored ribbons and d5 of TrkB is shown in gray molecular surface configuration. **(C)** Crystal structure of BDNF-NT4 dimer, PDB 1B8M. BDNF is shown in cyan and NT4 in green. The N-termini of both molecules are colored orange, and the C-termini are colored blue.

BDNF belongs to the classic neurotrophin family of trophic factors that also includes nerve growth factor (NGF), neurotrophin 3 (NT3) and neurotrophin 4 (NT4). Since the report of hippocampal BDNF and Tropomyosin receptor kinase B (TrkB) mRNA induction by electroconvulsive seizure and multiple classes of chronic chemical antidepressants (Nibuya et al., 1995), the interest in understanding the role of BDNF in antidepressant activity has continued to grow. The subsequent demonstration that a single infusion of BDNF into the hippocampus was independently capable of producing antidepressant-like effects, suggested a central role for BDNF in the therapeutic actions of antidepressants (Shirayama et al., 2002). There have since been numerous reports examining the role of BDNF in psychiatric disorders and it could emerge as a valuable biomarker in depression and treatment response (Polyakova et al., 2015) The neurobiology of BDNF and its role in antidepressant actions and psychiatric disorders are discussed in detail in two recent reviews (Castren and Monteggia, 2021; Wang et al., 2022). In this review we focus on signaling and structure. The finding of concomitant BDNF and TrkB regulation in the early report was fortuitous because TrkB, a receptor tyrosine kinase, is the high specificity receptor for BDNF. BDNF is produced by cells as a precursor protein, preproBDNF. Cleavage of the signal peptide results in proBDNF that can then be converted to mature BDNF. Both forms of BDNF are biologically active but bind to different receptors, proBDNF to p75^NTR^ and BDNF to TrkB, often resulting in opposing effects. Interestingly, an imbalance in levels of these two forms of BDNF has been reported in the serum of depressed patients, with decreased mature BDNF but not proBDNF (Yoshida et al., 2012). Our focus in this review is on mature BDNF. Dimeric BDNF binds to TrkB causing receptor dimerization leading to phosphorylation of cytoplasmic tyrosine residues in each other and an increase in kinase activity. The receptor kinases then phosphorylate additional tyrosine residues transforming them into attachment sites for phospholipase C (PLCγ1) and Shc (Figure 1A). PLCγ1 then catalyzes breakdown of lipids to generate diacylglycerol (DAG) and inositol trisphosphate (IP3) resulting in the release of calcium and activation of protein kinase C (PKC). Further downstream signaling activates mammalian target of rapamycin (mTOR) and cAMP response element binding protein (CREB), targets that have been strongly implicated in antidepressant activity (Blendy, 2006; Li et al., 2010).

Interestingly, both NT4 and BDNF bind to the same site on TrkB, the fifth extracellular domain (Figure 1B), either as homodimers or heterodimers (Robinson et al., 1999; Banfield et al., 2001). TrkB is 822 AA residues long, with 400 residues of the amino terminus comprising the extracellular domain which includes the BDNF and NT4 binding region, residues 286–383 (Robinson et al., 1999). A high-resolution crystal structure of BDNF homodimer bound to TrkB is unavailable and hence the NT4 and NT4-BDNF dimer structure is shown (Robinson et al., 1999). There is 70% similarity in the amino acid sequence of BDNF and NT4, but structurally they are more closely related with both comprising of eight β-strands organized into four antiparallel pairs of twisted β-strands (Figure 1C). Structural homology is high between the β-sheets of NT4 and BDNF (Robinson et al., 1999). Variation is mostly limited to the loop regions. The dimers are stabilized by hydrogen bonds and hydrophobic side-chain interactions between the β-strands. NT4 binds TrkB with a higher affinity (K_d_ of 260 pM) than BDNF (K_d_ of 790 pM; Banfield et al., 2001). However, the literature is rather sparse when it comes to the role of NT4 in antidepressant activity. Is this due to insufficient testing of NT4 in behavioral assays or a lack of antidepressant effects? It is useful to note that early work investigating hippocampal BDNF infusion also examined NT3 and NGF but not NT4 (Shirayama et al., 2002). While NGF lacked antidepressant activity, NT3 exhibited effects comparable to BDNF when infused into the dentate gyrus. The preferred high affinity receptor for NT3 is TrkC and for NGF it is TrkA. Despite the finding that both NT3 and BDNF bind the same active site on TrkB, it is likely that subtle differences in ligand-receptor interactions stabilize the dimerized receptor in different active conformations as in the case of biased agonism (Mohan et al., 2019). Recently, it was shown that antidepressants bind directly to the transmembrane helices of TrkB and conformationally position the dimers for BDNF signaling (Casarotto et al., 2021). This indicates that the conformational state of TrkB can determine the downstream signaling events responsible for antidepressant effects. Potential downstream molecules would include CREB, mTOR and calcium/calmodulin-dependent protein kinase II (CaMKII; Blendy, 2006; Li et al., 2010; Autry et al., 2011). mTOR, a serine/threonine kinase that integrates growth factor signals (including BDNF, IGF-1 and VEGF), nutritional status and cellular cues towards cell proliferation, circuit maintenance, plasticity, and regulation of behavior (Lipton and Sahin, 2014). It was shown to be centrally important in synaptogenesis and the antidepressant effects of ketamine (Li et al., 2010) and continues to investigated in the signaling pathway of novel compounds and prescription antidepressants (Xu et al., 2018; Neis et al., 2020). CaMKII, another serine/threonine kinase, phosphorylates several proteins involved in synaptic plasticity (Kim and Hayashi, 2014). It is also a key target of antidepressants and serves to transduce their molecular signaling actions (Barbiero et al., 2007; Adaikkan et al., 2018). In partnership with CaMKIV, it can regulate CREB, a transcription factor of major interest in antidepressant activity (Blendy, 2006; Yao et al., 2021). Activation of CREB would result in increased BDNF transcription and further amplify the cascade.

### Dysregulation of fibroblast growth factor 2 in depression and antidepressant effects

**Figure 2:**
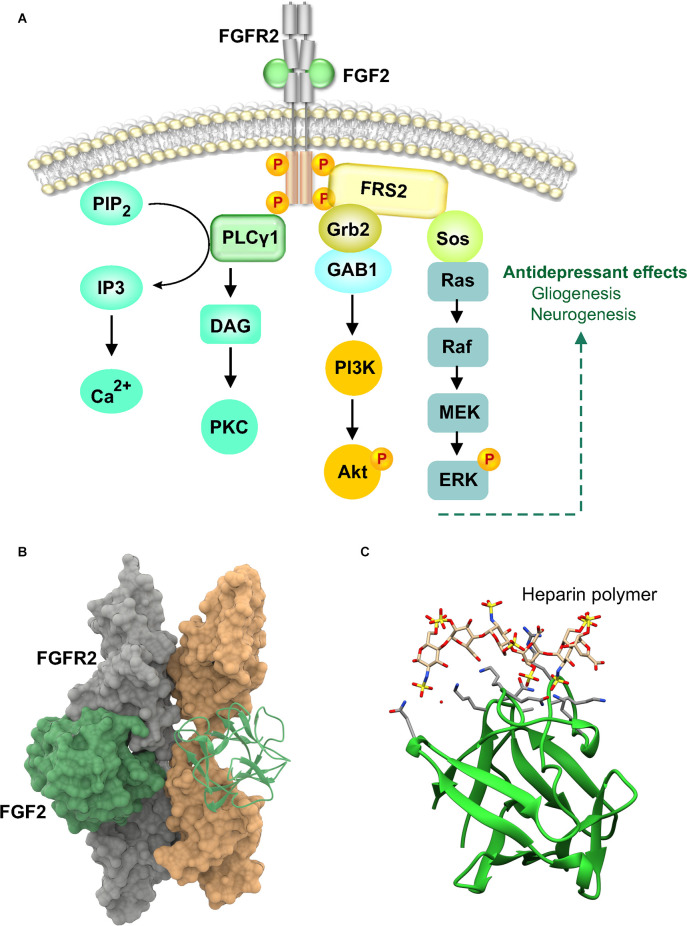
Fibroblast growth factor 2 (FGF2) signaling and structure. **(A)** FGF2 binds to FGFR2 at the D2-D3 extracellular domain and induces receptor dimerization which brings the intracellular tyrosine kinase domains into proximity to facilitate activation by transphosphorylation. Activated FGFR then phosphorylates other intracellular targets. The major one being FGFR substrate 2 (FRS2). FRS2 associates with PLCγ1 and growth factor receptor bound 2 (Grb2). Grb2 then recruits son of sevenless (SOS) or Grb2-associated binder 1 (GAB1). SOS activates the Erk cascade *via* Ras activation while GAB1 activates the PI3K-Akt pathway. Phosphorylation and activation of PLCγ1 by FGFR results in the catalysis of phosphatidylinositol-4,5 biphosphate (PIP2) to yield diacylglycerol (DAG) and inositol-1,4,5-trisphosphate (IP3). IP3 mobilizes calcium while DAG activates protein kinase C (PKC). **(B)** Two 1:1 FGF-FGFR complexes form a 2:2 dimeric structure without any direct FGF-FGF interaction (Plotnikov et al., 2000). The FGFR dimers are shown in the molecular surface configuration, PDB 1EV2. One FGF2 molecule is shown in the molecular surface configuration and the other as a ribbon structure. **(C)** Crystal structure of FGF2 consisting of 12 antiparallel β-strands is shown in ribbon representation with heparin, PDB 1BFC. Heparin-derived tetra and hexasaccharides are shown bound to FGF2. They bind to FGF residues asparagine-28, arginine-121, lysine-126, and glutamine-135. The binding site also includes lysine-27, asparagine-102, and lysine-136. Binding of heparin polymer does not alter FGF2 conformation and serves only to juxtapose ligand and receptor.

The fibroblast growth factor (FGF) family has 18 ligands that signal through four known tyrosine kinase receptors, fibroblast growth factor receptors, FGFR1–FGFR4 (Goetz and Mohammadi, 2013). Although there are 23 members in the FGF family, FGF11–FGF15 do not bind FGF receptors and are classified as FGF homologous factors due to high homology with the FGF family. The best studied FGF’s in the brain are FGF1 (also known as acidic FGF) and FGF2 (basic FGF). FGFs are the only growth factor that utilize an accessory molecule, heparin sulfate, for receptor signal transduction. Intriguingly, while FGF1 activity is enhanced 10–100-fold in the presence of heparin, FGF2 is relatively unaffected by heparin (Burgess and Maciag, 1989; Eckenstein et al., 1991). The roles of FGF in brain development have received substantial attention, demonstrating effects in migration, survival, cell fate and differentiation (Kuzis et al., 1995; Vaccarino et al., 1999a, b). A potential role for the FGF system in depression and antidepressant action was indicated by microarray gene expression analysis of postmortem frontal cortex brain tissue from major depressive disorder (MDD) patients, showing dysregulation of multiple FGF transcripts and a reduction in FGF2 (Evans et al., 2004). Reduced FGF2 expression in MDD was also reported in the hippocampus, but with an increase in FGF receptor 1 (Gaughran et al., 2006). It is interesting that a combination of fluoxetine and olanzapine, but neither drug independently, elevated FGF2 in rat brain (Maragnoli et al., 2004). Differential region-specific effects were observed as a single combined dose increased mRNA in the cortex, but chronic administration was required to increase hippocampal levels. This could be due to differences in brain region-specific receptor expression levels and/or sensitivity. Functional demonstration of antidepressant activity requires direct experimental evidence, which was obtained by intracerebroventricular (ICV) and prefrontal cortex (PFC) infusion of FGF2 (Turner et al., 2008; Elsayed et al., 2012). Furthermore, FGF2 is required for the antidepressant effects of fluoxetine (Simard et al., 2018). The rescue of stress-induced deficits in behavioral assays by FGF2 and the blockade of fluoxetine and imipramine’s antidepressant effects by FGFR1 inhibitor suggests that FGF signaling is important for antidepressant activity (Elsayed et al., 2012). A recent rat study implicated FGF2 signaling in the antidepressant effects of high-frequency repetitive transcranial magnetic stimulation, attaching additional significance to this pathway in the treatment of depression (Yan et al., 2022). From a cellular mechanism standpoint, FGF2 effects appear to be exerted *via* its role in supporting gliogenesis in the PFC (Elsayed et al., 2012) and neurogenesis in the hippocampus (Cheng et al., 2015). As a pleiotropic molecule, FGF2 also has potent effects on angiogenesis but this phenomenon is understudied in connection with antidepressant action. FGF2 signals by binding to FGFR and inducing receptor dimerization and facilitates transphosphorylation by bringing the kinase domains close to each other (Figure 2). This is followed by activation of a key molecule, FGF receptor substrate 2 (FRS2) and PLCγ1. Signaling further downstream FRS2 can occur either through phosphoinositide-3-kinase-protein kinase B (PI3K-PKB/AkT) or Ras-mitogen-activated protein kinase/extracellular signal-regulated kinase (MEK) pathways. It is likely that both these pathways are involved in the antidepressant actions of FGF2. Ras-MEK is a known mitogenic pathway and PI3K-AkT is well characterized in cell survival. As the behavioral effects produced by FGF2 are primarily *via* cellular mechanisms, it is unlikely to produce effects in a few days as seen with BDNF.

### Antidepressant-like activity of vascular endothelial growth factor

**Figure 3:**
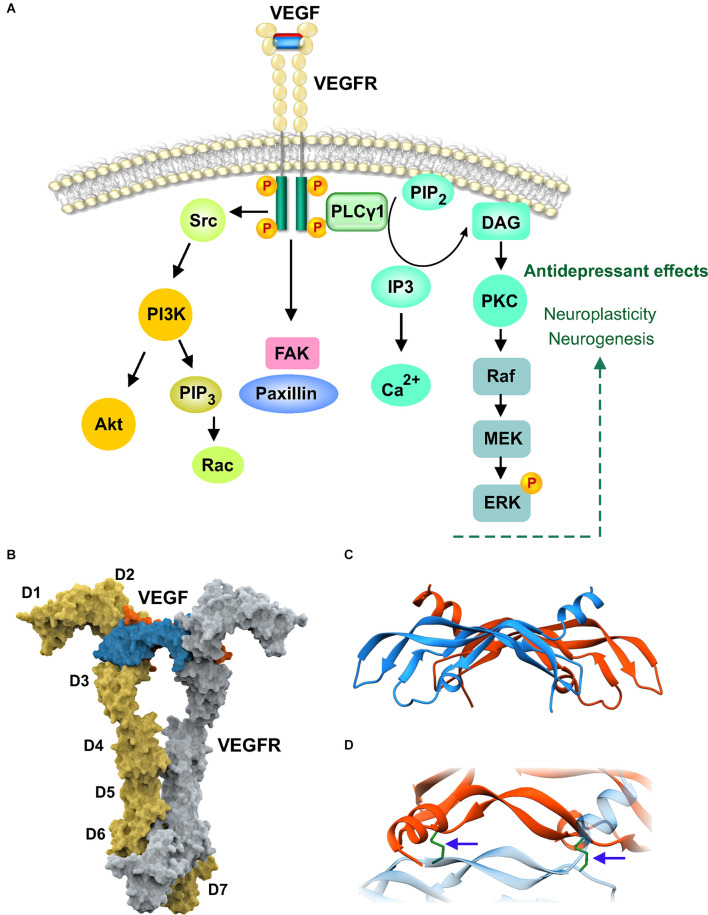
Vascularendothelial growth factor (VEGF) signaling and structure. **(A)** Extracellular and intracellular domains of dimerized vascular endothelial growth-factor receptor (VEGFR1) is shown bound to VEGF homodimer. VEGF binding causes dimerization and autophosphorylation of intracellular tyrosine residues. Intracellular proteins such as phospholipase C (PLCγ1) associate with the phosphorylated tyrosine residues *via* Src homology-2 (SH2) domains. PLCγ1 activation hydrolyzes membrane phosphatidylinositol-4,5 biphosphate (PIP2) to generate the second messengers diacylglycerol (DAG) and inositol-1,4,5-trisphosphate (IP3). DAG activates protein kinase C (PKC) while IP3 releases calcium stores. VEGFR also activates Src to trigger PI3K-Akt cascade and activates focal adhesion kinase (FAK) which associates with paxillin to mediate cell migration. **(B)** Crystal structure of the full-length extracellular domain of VEGFR1 in complex with VEGF, PDB 5T89. The receptor dimers are shown in the molecular surface representation in yellow and gray. The immunoglobulin (Ig) domains D1–D7 are indicated for one protomer. The VEGF dimer, in orange and blue, is shown in the molecular surface configuration bound to D2–D3. **(C)** Secondary structure of the VEGF dimer is shown in ribbon configuration, PDB 1FLT. **(D)** Magnified and rotated view of **(C)** showing the disulfide bonds (arrows) connecting the dimer chains.

VEGF has been heavily investigated in vascular biology due to its central role in regulating angiogenesis. VEGF-A, 121 AA in humans and 120 in mice, binds as a homodimer to cause receptor dimerization and activation of the intracellular kinase domain (Figure 3). The central structure consists of four antiparallel beta strands and a short helix close to the N-terminus. The homodimers are covalently connected by two disulfide bonds (Figure 3D). Larger VEGF isoforms contain additional amino acid residues, 145–206. Additional VEGF family members include VEGF-B, C, D and placental growth factor. The focus here is on VEGF-A and referred to as VEGF. The VEGF system includes three receptors, VEGFR1 or FMS-like tyrosine kinase 1 (Flt1), VEGFR2 or fetal liver kinase (Flk1), and VEGFR3 or Flt4. The VEGF receptor consists of seven Immunoglobin (Ig)-like domains with only the D2–3 domains involved in binding VEGF (Markovic-Mueller et al., 2017). Intertwining of the chains begins in D4–5 causing the lower domains to come into close contact (Figure 3B). Flk1 is the best characterized VEGF receptor and is responsible for signaling in both endothelial cells and neurons. This dual action of VEGF likely underlies its neurogenic effects in the hippocampal subgranular zone as neurogenesis occurs in synchrony with angiogenesis (Palmer et al., 2000). Interestingly, intracerebroventricular VEGF infusion produced antidepressant-like effects in rodent models and signaling *via* Flk1 was necessary for the neurogenic actions of multiple classes of antidepressants (Warner-Schmidt and Duman, 2007; Segi-Nishida et al., 2008). A partial rescue of behavioral deficits by VEGF was noted in the offspring of a maternal immune activation model (Sideromenos et al., 2020). VEGF has been shown to be necessary for antidepressant actions of BDNF that are mediated *via* the mPFC, raising the possibility that downstream molecules that modulate antidepressant effects are shared by trophic factor signaling pathways (Deyama et al., 2019). The norepinephrine and serotonin systems have also been implicated in the antidepressant actions of VEGF, potentially indicating cross talk between growth factor and neurotransmitter signaling (Udo et al., 2014). If the antidepressant mechanism of VEGF is predominantly neurogenic, it would not be surprising for it to play a limited role in the actions of a fast-acting drug such as ketamine (Choi et al., 2016). However, VEGF has also been shown to enhance neuroplasticity and improve memory function independent of neurogenesis and angiogenesis (Licht et al., 2011). A caveat with developing VEGF as a drug to modulate mood is its known effects on increasing vascular permeability. It is for this reason that VEGF was initially named as vascular permeability factor (VPF). Higher doses can therefore adversely influence blood-brain barrier integrity. Can the vascular permeability effects of VEGF be disassociated from the neuronal actions that influence behavior in psychiatric disease? As the vascular and neuronal actions are produced by signaling through the same receptor, but on different cell types, it would be quite challenging to obtain neuron-selective receptor signaling.

### Systemic insulin-like growth factor 1 produces antidepressant effects

**Figure 4:**
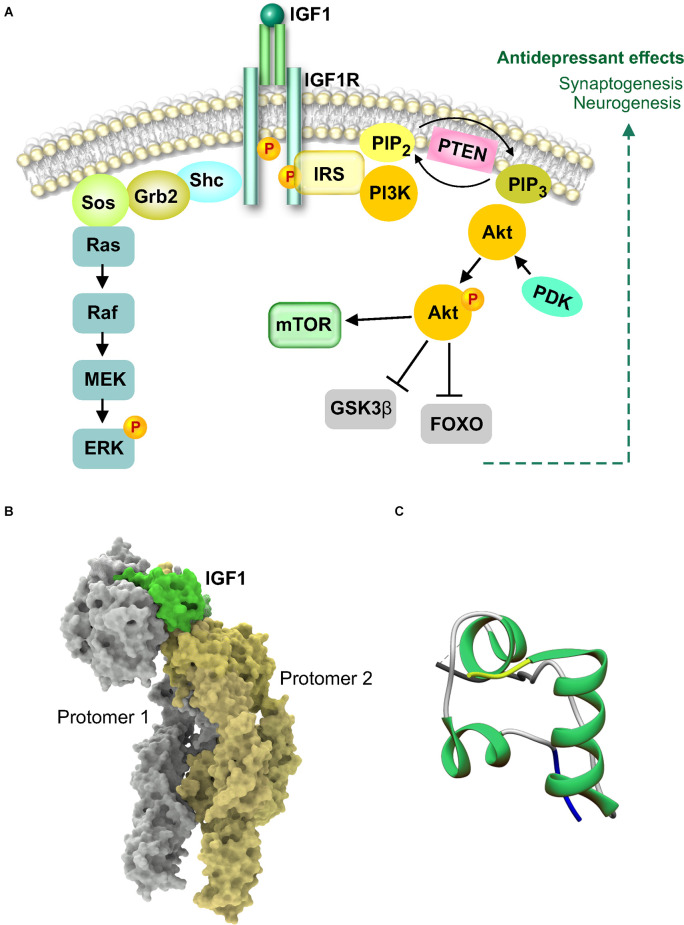
Insulin-like growth factor 1 (IGF-1) signaling and structure. **(A)** IGF1 binds as a monomer to the extracellular domain of IGF1 receptor (IGFR1) to cause autophosphorylation and activation of the intracellular tyrosine kinase domain. Activation of IGF1R causes association with insulin receptor substrate (IRS) docking protein. IRS can associate with Shc and Grb2 to activate the Ras-MAPK pathway or activate PI3kinase. Activated PI3K and the generation of phospholipids results in activation of Akt leading to phosphorylation and inhibition of GSK3β. Activated Akt can also activate mammalian target of rapamycin (mTOR). **(B)** Cryo-electron microscopy structure of the full-length extracellular region of the IGF1R-IGF1 active complex, PDB 6PYH. The structure shows one IGF1 molecule bound to asymmetric IGF1R. IGF1 and the receptor dimer are shown in the molecular surface configuration. **(C)** X-ray diffraction crystal structure of IGF1 showing the N-terminus in yellow and the C-terminus in blue (Vajdos et al., 2001), PDB 1IXM.

The mammalian insulin growth factors have been well studied in growth, development, and body size regulation. The induction of IGF-1 at brain injury sites suggested that it could be part of a protective or repair response involved in neuroprotection (Gluckman et al., 1998; Carro et al., 2001). As systemic administration was sufficient to increase adult hippocampal neurogenesis, IGF-1 became an attractive molecule to investigate in neuropsychiatry (Aberg et al., 2000). Unlike other trophic factors that exhibit limited transport across the BBB from the periphery, the brain is a target organ for serum IGF-1, with transport occurring primarily *via* the choroid plexus (Carro and Torres-Aleman, 2006). IGF-1 is a 70 amino acid molecule consisting of three helices which binds to the IGF-1 receptor (IGFR1), a class II receptor tyrosine kinase from the insulin receptor family, as a monomer (Li et al., 2019; Figure 4). IGF-1 binding to the receptor dimer produces a major conformational change that results in the asymmetric structure shown in Figure 4B. The activation of tyrosine kinase activity causes phosphorylation of substrate molecules, insulin receptor substrate (IRS) and Src homology collagen (Shc). The phosphorylated substrates are recognized by Grb2 and the regulatory subunit of PI 3-kinase. Further downstream signaling can occur *via* multiple pathways, Akt-mTOR, ras-raf-MEK-ERK, and Jak-STAT (Himpe and Kooijman, 2009; Dyer et al., 2016). An interesting intertwining of trophic factor and neurotransmitter receptor signaling was shown by the hippocampal upregulation of IGF-1 in response to a serotonin type 3 receptor (5HT3R) agonist (Kondo et al., 2018). The antidepressant effects of 5HT3R agonism occurred *via* IGF-1 mediated neurogenesis and was independent from fluoxetine-induced mechanisms, potentially offering an alternate pathway for antidepressant activity in SSRI-resistant patients (Kondo et al., 2018). Apart from a neurogenic mechanism of action, IGF-1 is also involved in the rapid as well as sustained effects of ketamine (Deyama et al., 2022). These recent studies show that the antidepressant-like effects of ketamine that require IGF-1 release from the medial prefrontal cortex, are independent of BDNF but likely involve mTOR (Deyama et al., 2022). The dissociation of BDNF’s involvement in ketamine’s actions is intriguing and it will be interesting to learn from future studies regarding the status of TrkB activation when a BDNF neutralizing antibody is used as opposed to BDNF deletion.

In contrast to other trophic factors which are delivered directly to the brain due to their inability to cross the blood-brain or blood-CSF barrier, IGF1 produces antidepressant-like effects after peripheral administration (Duman et al., 2009). The ability of peripheral IGF1 to activate signal transduction in the brain could underlie the key role it plays in the behavioral and neurogenic effects of physical exercise (Cotman et al., 2007; Duman et al., 2009). Pharmacological inhibitor studies confirmed that brain IGFR1 receptors mediated the antidepressant effects of IGF1 (Burgdorf et al., 2015; Mueller et al., 2018). As the levels of free IGF1 are regulated by specific IGF binding proteins (IGFBPs), antidepressant effects can be produced by inhibiting IGFBP association with IGF and thereby elevating the levels of IGF1 available for receptor binding (Malberg et al., 2007). This provides an alternate approach whereby endogenous IGF-1 can be manipulated to produce a therapeutic response. The IGF-1 system is vulnerable to early stress exposure and continues to be downregulated in adult animals that experienced prenatal stress (Basta-Kaim et al., 2014a, b; Trojan et al., 2016). Precisely how this occurs is currently unknown but could involve reduction in ERK signaling and CREB activity (Guan et al., 2013). In a field desperately in need of reliable biomarkers, IGF-1 could emerge as a stress vulnerability biomarker given the promising results from rodent and large human population studies (Santi et al., 2018). Whether the results from preclinical studies of IGF-1-induced antidepressant actions can translate to the clinic are yet to be seen. It is worth noting that among the trophic factors discussed in this review, IGF-1 is the smallest in size at 70 AA. This could aid its candidacy for intranasal administration in a manner comparable to insulin (Craft et al., 2020). If successful, this mode of administration can reduce the potential for off-target systemic effects, particularly when chronic dosing is required as is likely to be the case in depression. In designing such studies, it is important to consider that trophic factors are potent molecules and can produce adverse effects, including seizures, when they exceed physiological levels in particular brain sub-regions and circuits (Scharfman, 2005).

### Erythropoietin (EPO) exhibits antidepressant effects in rodents and humans

**Figure 5:**
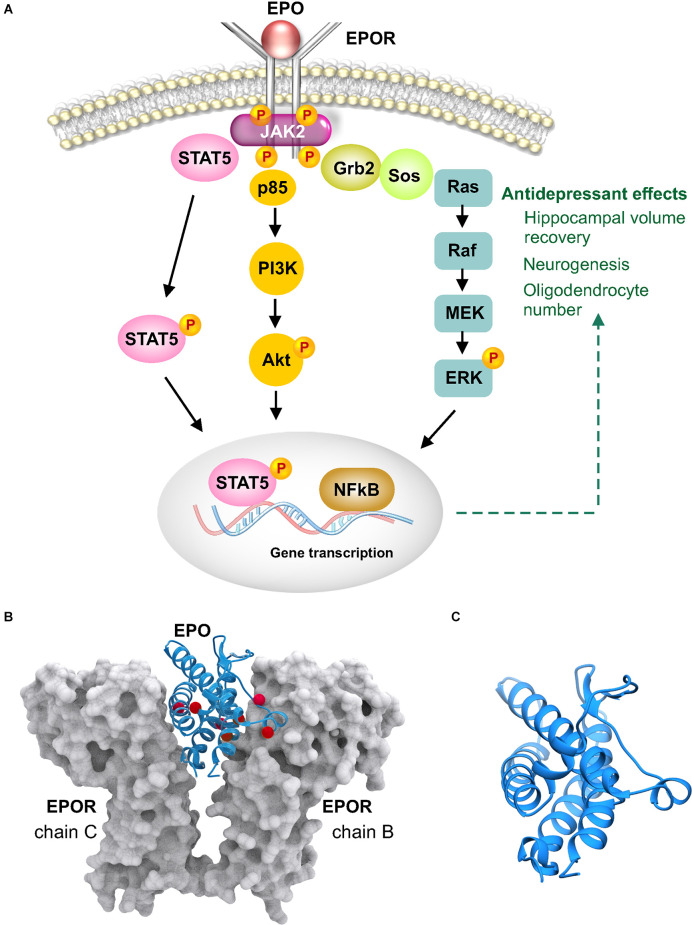
Erythropoietin (EPO) signaling and structure. **(A)** EPO binds as a monomer to dimerized erythropoietin receptor (EPOR) causing conformational change that results in the intracellular region associating with janus kinase 2 (JAK2). JAK2 phosphorylates EPOR and facilitates recruitment of intracellular proteins such as growth factor receptor bound 2 (Grb2) and signal transducer and activator of transcription (STAT). Phosphorylated EPOR also activates the PI3K-Akt signaling cascade. **(B)** Crystal structure of EPO bound to EPOR, PDB 1EER. EPO is shown in blue ribbons and EPOR in gray molecular surface configuration. Shown as red spheres are residues that are carbamoylated in non-erythropoietic carbamoylated EPO (Sathyanesan et al., 2018). **(C)** EPO is shown magnified revealing the four α helices in an up-up-down-down arrangement.

EPO belongs to the class I cytokine family and is well studied and widely prescribed for the treatment of anemia. EPO binds as a monomer to preformed receptor dimers producing a striking change in the conformation of the dimers resulting in an asymmetric binding to the receptors (Syed et al., 1998; Remy et al., 1999; Figure 5B). EPO is a 166 AA protein consisting of four α helices arranged in an up-up -down-down topology (Figure 5C). In contrast to the other receptors discussed earlier, the erythropoietin receptor (EPOR) does not possess autocatalytic activity and relies on its association with Janus kinase 2 (JAK2) to phosphorylate EPOR’s intracellular domain. The phosphorylated residues serve as docking sites for SH2 domain containing proteins such as the transcription factors from the signal transducer and activator of transcription (STAT) family (Figure 5A). Further downstream signaling can occur *via* three main cascades, Jak-STAT, PI3K-Akt, and MAPK-Erk (Constantinescu et al., 1999). The Jak-STAT pathway is widely accepted as the canonical signaling cascade for erythropoietic activity (Richmond et al., 2005).

When studies began reporting on the CNS effects of EPO in the late 1990s, it was already an FDA approved (in 1989) anemia drug (Genc et al., 2004; Brines and Cerami, 2005). The demonstration of neuroprotective (Viviani et al., 2005), neurogenic (Lu et al., 2005) and neurotrophic (Siren et al., 2001) effects and the fact that systemic administration was sufficient for CNS effects (Brines et al., 2000), made it a promising candidate to test the neurotrophic hypothesis of depression in human studies. The antidepressant-like actions were demonstrated in both mice and rats (Girgenti et al., 2009; Osborn et al., 2013). Human studies in normal volunteers and depressed patients indicated that the antidepressant effects of EPO were comparable to prescription antidepressants (Miskowiak et al., 2007b, Miskowiak et al., 2010). Interestingly, the effects were discernible as early as 3 days after EPO administration (Miskowiak et al., 2008). Neurobiological insight regarding the mechanism of action was obtained from imaging studies indicating that the hippocampus is a site of action (Miskowiak et al., 2007a). The reversal of volumetric loss in the CA1–CA3 region of the left hippocampus in treatment resistant depressed patients suggests that with an extended dosing regimen EPO produces cellular effects that are likely different from those that occur at 3 days after a single dose (Miskowiak et al., 2015). These results are of major importance to the field as a reduction in hippocampal volume in depression has been reported by multiple groups (Sheline et al., 1996; Bremner et al., 2000; Videbech and Ravnkilde, 2004). The cellular phenomenon or the phenotype of cells involved in the volumetric loss are yet to be conclusively identified. Our understanding of disease pathophysiology can therefore be enhanced by obtaining further insight regarding the cellular changes produced by EPO. It is in this context that results from preclinical studies of EPO, reporting an approximately 20% increase of oligodendrocytes and pyramidal neurons in the hippocampus, are rather intriguing (Hassouna et al., 2016; Wakhloo et al., 2020). Developing these findings has the potential to bridge the knowledge gap between preclinical and clinical results regarding neurotrophic mechanisms that are driven by trophic factors in neuropsychiatry.

Although promising behavioral effects of EPO have been demonstrated in rodents and humans, its inherent erythropoietic activity can produce adverse effects in non-anemic individuals by supraphysiological elevation of red blood cell counts. The discovery of carbamoylated erythropoietin (CEPO), a chemically engineered non-erythropoietic derivative of EPO, was therefore a significant advancement as it dissociated the neurotrophic activity of EPO from the erythropoietic effects (Leist et al., 2004). Demonstration of antidepressant-like activity provided additional support for neurotrophin-mediated behavioral response (Leconte et al., 2011; Sampath et al., 2020). The antidepressant-like effects of CEPO can involve neurogenesis as in the case of the Leconte et al. study which used chronic administration for over 6 weeks (Leconte et al., 2011), as well as neurogenesis-independent behavioral effects requiring only four doses (Sampath et al., 2020). Interestingly, four doses of CEPO were sufficient to produce effects in the novelty-induced hypophagia test, an assay that is sensitive to chronic but not acute administration of fluoxetine (Dulawa and Hen, 2005). Non-erythropoietic EPO derivatives that are behaviorally active are potential templates for safely harnessing the neuronal effects of EPO into the development of neurotrophic drugs (Pekas et al., 2020).

### The questions of specificity and non-specificity

There are multiple angles from which to examine the role of specificity vs. non-specificity in trophic factor signaling. From a treatment modality standpoint, physical exercise and electroconvulsive seizure are known to activate several trophic factor cascades, including those discussed in this review (Newton et al., 2003; Altar et al., 2004; Cotman et al., 2007; Hunsberger et al., 2007; Girgenti et al., 2009). Does the simultaneous activation of several trophic cascades play a role in their antidepressant activity? Studies that have tested individual trophic factors, such as VGF (non-acronymic) in exercise, have shown that although several neurotrophins, including BDNF, are regulated by exercise, administration of VGF alone was sufficient to elicit antidepressant-like effects in mice (Hunsberger et al., 2007). Given BDNF’s well documented antidepressant effects, a key question in this regard is whether VGF can be antidepressant in the absence of BDNF signaling. Available evidence indicates that VGF likely needs BDNF as they act in concert with TrkB act by functioning in an autoregulatory loop to produce antidepressant effects (Jiang et al., 2019). It would be worthwhile to investigate whether there are comparable relationships among other trophic factors and how they modulate signaling interactions and crosstalk.

Despite substantial structural diversity among trophic factors and the receptors that they bind, there is significant overlap in the signaling pathways that they activate (Chao, 1992). On the other hand, in neurotrophic factor classes with high homology such as among BDNF, NT3, NT4 and NT5, antidepressant activity is mostly limited to BDNF and TrkB. A key question in this context pertains to the essential downstream molecules for antidepressant activity, or is it a particular molecular signaling configuration of activated and inactivated signaling molecules? The selectivity of signaling and cellular responses of the different classical neurotrophins that act *via* Trk receptors was rigorously addressed and yielded six lines of evidence (Segal, 2003). (1) The Trks differentially activate Akt and Raf, (2) ligand binding is associated with signaling specificity, (3) differences in the tempo of receptor stimulation can determine short-term vs. long-term effects, (4) location of receptors, pre or postsynaptic, (5) ability of non-neurotrophic ligands such as adenosine to influence Trk signaling, and (6) the preferential use of particular adaptor and signaling molecules, for example different Shc, Ras, and PI3K catalytic isoforms. While the above points can explain the specificity of trophic factor signaling and shed light on why closely related neurotrophins produce differential behavioral effects, it remains to be seen if structurally unrelated trophic factors binding to diverse receptors produce antidepressant effects due to an overlap in downstream signaling.

In this regard, it is important to note that it is the contribution of trophic signaling to neuronal structure and function in key brain circuits that eventually produces changes in behavior. The cellular phenomena that they regulate such as neurogenesis, gliogenesis and synaptogenesis are important determining factors as to whether the effects are long lasting or short term. There are however questions that can only be answered by further research. As shown in multiple instances, blockade or inhibition of trophic signaling (BDNF, VEGF, FGF) results in a loss of the antidepressant effects of conventional antidepressants. Why does the inhibition of one pathway truncate behavioral effects? Perhaps this is due to crucial common targets downstream trophic cascades that are subject to control upstream. Can inhibition of trophic signaling in non-depressed individuals lead to depression? Several tyrosine kinase inhibitors are in use to block growth factor signaling in cancer (Arora and Scholar, 2005) and it is therefore important to know if they cross into the brain and impact neuronal trophic signaling. An increased incidence of anxiety and depression was reported in patients receiving anti-VEGF treatments (van der Aa et al., 2021). Interestingly, discontinuing tyrosine kinase inhibitor treatment has also shown to precipitate depression (Sogawa et al., 2018). Preclinical studies will be necessary to understand the relationship between the long-term consequences of inhibiting specific growth factor signaling receptors and brain health.

Neurotrophic factor receptor signaling continues to be an important research focus in psychiatric neuroscience due to its mechanistic role in antidepressant action, potential for neurotrophic drug development and importance in brain development and function. Future work aimed at methods to preclude undesirable off target systemic effects while safely harnessing the neurotrophic and behavioral actions, can enable clinical application of these molecules in the context of neuropsychiatric diseases.

## Author Contributions

MS conducted literatures searches and provided critical intellectual input. SN wrote the manuscript and prepared the figures. All authors contributed to the article and approved the submitted version.

## Funding

This work was supported by U.S. Public Health Service (National Institutes of Health), grant MH106640 (SN) and the University of South Dakota Center for Brain and Behavioral Research. The funding agency had no role in writing of the manuscript.
